# Exploring the Antimicrobial Potential and Cytotoxic Effects of Different Brassica oleracea Varieties

**DOI:** 10.7759/cureus.59613

**Published:** 2024-05-03

**Authors:** Kavishri S, Suganya Panneer Selvam, Rajeshkumar Shanmugam, Ramya Ramadoss, Sandhya Sundar, Pratibha Ramani

**Affiliations:** 1 Oral Biology, Saveetha Dental College and Hospitals, Saveetha Institute of Medical and Technical Sciences, Saveetha University, Chennai, IND; 2 Nanobiomedicine Lab, Centre for Global Health Research, Saveetha Dental College and Hospitals, Saveetha Institute of Medical and Technical Sciences, Saveetha University, Chennai, IND

**Keywords:** cytotoxicity, antifungal, antimicrobial, caries, health, dental, cabbage

## Abstract

Introduction: Dental caries has become a substantial global health burden, and many techniques have been used in dentistry to protect the tooth from decay. *Brassica oleracea* is a green cruciferous vegetable with a good source of vitamins C, K and E, which are also effective antibiotics and antioxidants. These characteristics will shield the oral cavity from pathogenic onslaught and can be considered during the formulation of antimicrobial mouthwash, toothpaste, or dental sealants.

Materials and methods: *B. oleracea* extract was prepared by heating and condensing the red and green cabbage. Both extracts were assessed for antimicrobial activity (antibacterial and antifungal activities) and cytotoxicity. After incubation, the zone of inhibition was calculated for antibacterial activity and the number of live nauplii for cytotoxicity.

Results: The extract from red cabbage was found to have more effective antibacterial properties than that from green cabbage. The red *B. oleracea* extract formed the highest zone of inhibition against *Candida albicans* (20 mm), followed by *Enterococcus faecalis* (15 mm) and *Streptococcus mutans* (9 mm). In contrast, the green cabbage extract formed the highest inhibition against *E. faecalis* (12 mm). The cytotoxicity increases with increased concentration, with the highest toxicity at 20 µL for both extracts.

Conclusion: The properties of *B. oleracea* can be utilized in dental products such as toothpaste, mouthwash, and dental sealants due to their antibacterial effects. By incorporating *B. oleracea* extracts into these products, oral health professionals may soon have additional tools to promote oral hygiene and prevent oral infections, offering a natural and effective alternative to traditional oral care ingredients.

## Introduction

Dental caries is characterized by the demineralization of the inorganic component of the tooth structure, primarily hydroxyapatite crystals, due to the acidic byproducts produced by bacteria in dental plaque. It affects the quality of life as it might remain the focus of infection and exacerbate or induce systemic disease. It peaked in the 19th and 20th centuries as a result of the increased availability of sugar in many developed nations. Though the invention of fluoride in the early 1970s decreased the rate of dental caries, its prevalence currently has increased due to food habits and lifestyle [1]. There are around 2.44 billion people worldwide suffering from permanent tooth decay, and 486 million children suffer from dental caries of the primary teeth [2]. Factors contributing to the prevalence of dental caries include socioeconomic status, educational level, oral hygiene practices, dietary habits, and gender. For instance, the consumption of sugary foods, poor toothbrushing habits, and lack of awareness about dental caries have been identified as significant contributors to the increasing prevalence of dental caries in developing countries [3]. Dental caries, a bacterial infection in the oral cavity, causes an acid attack that leads to tooth damage. However, the process of demineralization and remineralization is partly regulated by the presence of calcium and phosphate ions in saliva [4,5].

Dental caries is a significant public health concern with far-reaching impacts on society. The World Health Organization (WHO) has set global targets to develop cost-effective community-based methods to decrease dental caries as a large proportion of dental caries remain untreated due to high economic demands [6,7]. This goal can be achieved by implementing a range of measures, such as imposing a tax on sugar-sweetened beverages, fluoride application, and regular checkups. Our objective is to produce an affordable product with strong antibacterial properties to minimize the incidence of dental caries.

The sealants and mouth rinses are mostly bactericidal and bacteriostatic against *Streptococcus mutans*, the most common bacteria that initiate dental caries [8]. The main disadvantages reported are changes in taste sensation, tooth stains, burning sensation, incomplete photopolymerization, microleakage, and bond strength [9]. The herbal concoctions are thought to be moderately effective and less harmful than the majority of commercially available pharmaceutical mouthwashes [10]. We chose the Brassica family, specifically *B. oleracea*, as it contains polyphenols, vitamins (C, K, and E), minerals (selenium, iron, and zinc), secondary metabolites, and fiber [11]. We aimed to evaluate and compare the antibacterial properties and cytotoxicity between red and green *B. oleracea*.

## Materials and methods

Preparation of cabbage extract

**Figure 1:**
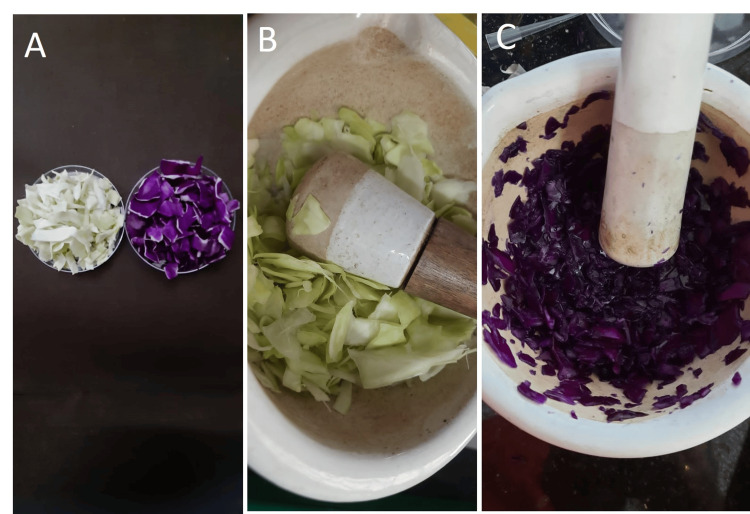
Preparation of the B. oleracea extract. (A) Green and red cabbage. (B,C) Milling

**Figure 2:**
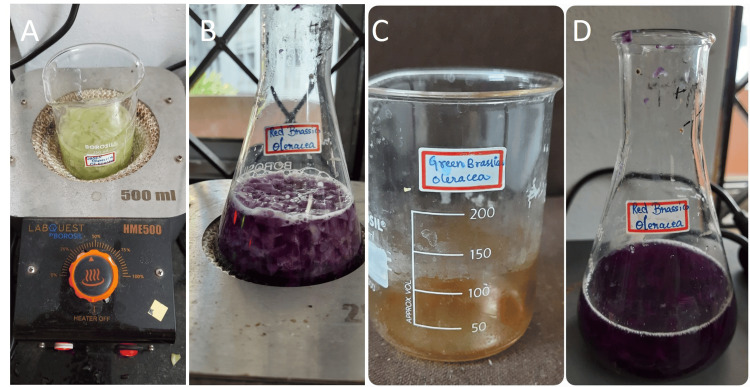
(A,B) Condensation and (C,D) filtration and storage of the B. oleracea extract

The red and green samples of *B. oleracea* were obtained from organic farms in Poonamallee, Chennai, Tamil Nadu, India. They were thoroughly washed with tap water, cut into small pieces, and then dried in the shade for four to five days before being finely powdered (Figure 1). Distilled water of 100 mL was added to 50 g of the powder extract. The mixture was heated between 60°C and 80°C for 10 minutes before being filtered through Whatman filter paper. Red and green cabbage extracts were filtered, then centrifuged at 5000 rpm, and refrigerated at 4°C (Figure 2).

Antimicrobial activity

Antibacterial Activity

Using Mueller-Hinton agar, the extract's antibacterial activity was assessed against the strains of *S. mutans*, *Enterococcus faecalis*, and *Staphylococcus aureus* to determine the zone of inhibition. The agar medium was produced, sterilized, and allowed to harden for 16 minutes at 121°C. The test organisms were swabbed after the wells were cut with a 9-mm sterile polystyrene tip. The extract was loaded in various concentrations (5, 10, 15, and 20 µL), and the zone of inhibition was evaluated following the plates' 24-hour incubation at 37°C. 

Antifungal Activity

*Candida albicans* activity was detected using the agar-well diffusion assay method with rose bengal agar as a medium. Before different amounts of the extract (5, 10, 15, and 20 µL) were added to the wells, the sterile medium, which was prepared in advance, was swabbed with the test pathogen. For 40-72 hours, the plates were incubated at 37°C. After the incubation time, the zone of inhibition was evaluated.

Cytotoxicity activity

Brine Shrimp Lethality Assay

The brine shrimp lethality assay is a bioassay used to evaluate the toxicity of chemical compounds or natural extracts. This method is simple and cost-effective for screening the toxicity of compounds and extracts, with the advantage of using a live organism as an indicator of toxicity. The hatching solution is the seawater. The solution was created by weighing and dissolving iodine-free salt in 200 cc of distilled water. The six-well culture plates were filled with 10-20 mL of saline water. Ten nauplii were added to each well (5 µL, 10 µL, 20 µL, 30 µL, 40 µL, 80 µL, and control). The required concentrations of the extracts were then added. The plates were incubated for 24 hours. The culture plates were examined after 24 hours to count the live nauplii present and were then computed using the following formula: (number of dead nauplii)/(number of dead nauplii + number of live nauplii) × 100.

## Results

Comparison of antimicrobial activity of the extract

**Figure 3:**
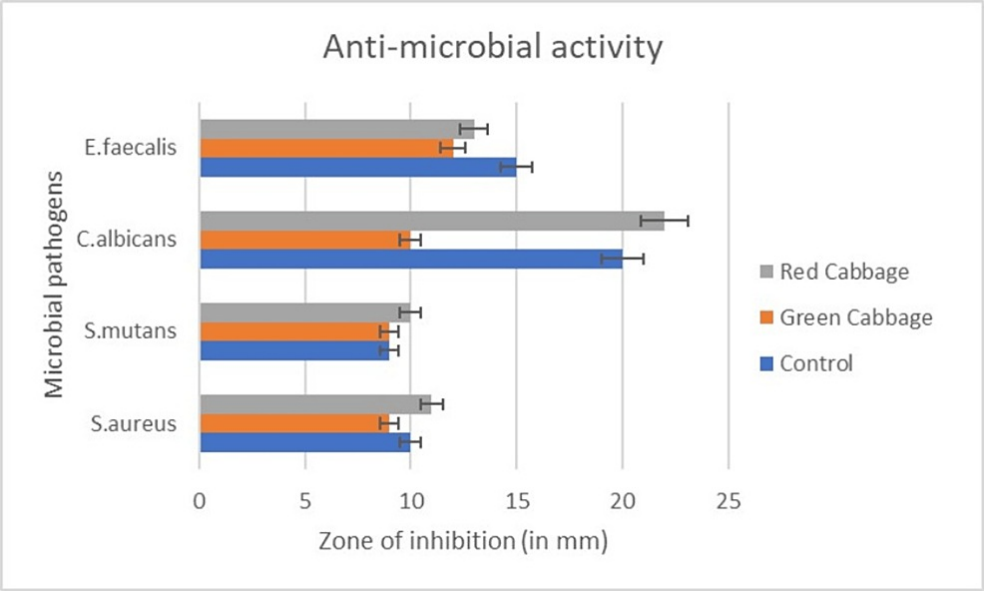
Comparison of antimicrobial activity of the red and green B. oleracea against oral pathogens with the highest activity against C. albicans with 20 mm of zone of inhibition

The red cabbage extract and the green cabbage extract exhibited the same antimicrobial properties against *S. mutans* at 100 µL, with a 9-mm zone of inhibition for both; the zone of inhibition for *S. aureus* at 100 µL was 10 mm for red cabbage and 9 mm for green cabbage; and the zone of inhibition for *E. faecalis* at 100 µL was 15 mm for red cabbage and 12 mm for green cabbage (Figure 3). The highest zone of inhibition of red cabbage and green cabbage was found against *C. albicans*, and it was 20 and 10 mm, respectively, at 100 µL (Table 1 and Figure 3). The control used for the antibacterial activity was streptomycin and fluconazole for antifungal activity.

**Table 1:** Comparison of antimicrobial activity of the red and green B. oleracea against oral pathogens

Organism name	Red cabbage (mm)	Green cabbage (mm)
S. aureus	10	9
S. mutans	9	9
C. albicans	20	10
E. faecalis	15	12

Comparison of cytotoxicity of the extract within 24 hours

**Figure 4:**
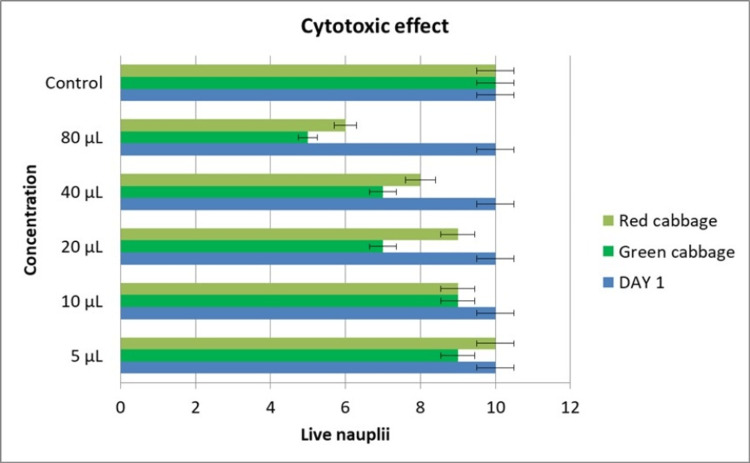
Comparison of cytotoxic activity of the red and green B. oleracea at various concentrations within 24 hours

The cytotoxicity of the extract was found with the number of live nauplii after 24 hours with various concentrations. The cytotoxicity was increased with increased concentrations. The number of live nauplii at 5 µL for red and green cabbage was ten and seven, which decreased to six and five at 20 µL when compared with the control (Table 2 and Figure 4).

**Table 2:** Comparison of cytotoxic activity of the red and green B. oleracea at various concentrations

Concentration	Number of live nauplii in green cabbage	Number of live nauplii in red cabbage
5 µL	7	10
10 µL	7	6
20 µL	5	6
Control	10	10

## Discussion

The Brassicaceae family consists of broccoli, cabbage, and kale, which are excellent sources of health-promoting phytochemicals and secondary metabolites, especially glucosinolates [12]. Many studies had been conducted with the genre Brassica to identify the antimicrobial effect; however, we attempted to identify the antimicrobial property of the extracts from the red and green cabbage in a biological way without adulteration of any other substances and compared the antibacterial and antifungal properties among them [13,14]. Silver nanoparticles synthesized from broccoli were found to be effective against pathogens such as *E. coli, S. mutans, Staphylococcus epidermidis*, and *Pseudomonas aeruginosa* [15].

Among the four varieties of *B. oleracea*, broccoli and red cabbage extracts showed the highest antimicrobial activity against *E. coli*, which was consistent in our study and resistant to *Salmonella typhimurium* [16]. Broccoli sprouts exhibited antibacterial activity against *S. aureus* and *E. coli*, similar to our results derived from the cabbage extract [17].

The antimicrobial activity of red and green cabbage was evaluated, and our results concluded that red cabbage had very effective antimicrobial properties and lower cytotoxicity compared to green cabbage. The extract from red cabbage showed the highest zone of inhibition against *C. albicans*, while the extract from green cabbage showed the highest zone of inhibition against *E. faecalis*. The cytotoxicity of both extracts increased with an increase in concentrations, with the highest cytotoxicity noted at 20 µL for the extract from green cabbage, and the least cytotoxicity was observed at 5 µL, which was equivalent to the control.

The antimicrobial peptides such as defensins, thionins, and proline-rich antimicrobial peptides were responsible for the antibacterial properties in *B. oleracea* [18]. The antimicrobial activity of the biosynthesized silver nanoparticles from *B. oleracea* showed the highest zone of inhibition formed against *Klebsiella pneumoniae,* followed by *E. coli and* *Staphylococcus saprophyticus* [19]. The ethanolic crude extract obtained from *B. oleracea* var. acephala exhibited significant antimicrobial activity, displaying a prominent zone of inhibition against *E. coli*, *S. aureus*, and *P. aeruginosa* [20]. The extract from red cabbage showed stronger antibacterial effects in our study. 

The extract from red cabbage showed the highest zone of inhibition against the *C. albicans* strain, possibly due to the presence of antifungal peptides such as sulforaphane, organic acids such as ascorbic acid, and phenolic compounds such as ferulic acid and caffeic acid in *B. oleracea*. Broccoli has antifungal properties because of the presence of an element called rutin [21]. The sulforaphane, which is converted from glucoraphanin in the broccoli, was found to have both blocking and suppressing action against the cancer stem cells; thus, it can also be added in the mouthwash prescribed for oral cancer or oral potentially malignant disorders where the conversion of procarcinogen to a carcinogen was blocked by the sulforaphane [22]. Though there were many studies regarding the antibacterial, antifungal, and antimicrobial activities of the Brassica genre, only broccoli was used, and comparative analyses were not done. We used red and green cabbage to compare their antimicrobial activity and found that red cabbage was more potent.

Limitations of the study

The present study focused on in vitro evaluations. Future studies should demonstrate in vivo and clinical trials to evaluate the safety of the prepared extract. Additional studies will be done to formulate the specific concentration with maximum benefits.

## Conclusions

Dental caries is still considered a global burden. To achieve the future goals of WHO, we are in a place to take steps to produce cost-effective mouthwash, pit and fissure sealants, and toothpaste to prevent dental caries. This research introduces a simple and environment-friendly synthesis of extract from red and green cabbage, which is antibacterial and antifungal. Our future research will involve identifying the compounds responsible for the medicinal properties and manufacturing an affordable product to prevent or minimize dental caries. This type of green synthesis strategy will open eco-friendly new horizons, contributing to the development of newer solutions to address global challenges in the health sector and its impact on the environment.
